# Evaluation of the 2023 Duke-International Society of Cardiovascular Infectious Diseases Criteria in a Multicenter Cohort of Patients With Suspected Infective Endocarditis

**DOI:** 10.1093/cid/ciae039

**Published:** 2024-02-08

**Authors:** Matthaios Papadimitriou-Olivgeris, Pierre Monney, Michelle Frank, Georgios Tzimas, Piergiorgio Tozzi, Matthias Kirsch, Mathias Van Hemelrijck, Robert Bauernschmitt, Jana Epprecht, Benoit Guery, Barbara Hasse

**Affiliations:** Infectious Diseases Service, Lausanne University Hospital and University of Lausanne, Lausanne, Switzerland; Department of Cardiology, Lausanne University Hospital and University of Lausanne, Lausanne, Switzerland; Department of Cardiology, University Hospital Zurich and University of Zurich, Zurich, Switzerland; Department of Cardiology, Lausanne University Hospital and University of Lausanne, Lausanne, Switzerland; Department of Cardiac Surgery, Lausanne University Hospital and University of Lausanne, Lausanne, Switzerland; Department of Cardiac Surgery, Lausanne University Hospital and University of Lausanne, Lausanne, Switzerland; Department of Cardiac Surgery, University Hospital Zurich and University of Zurich, Zurich, Switzerland; Department of Cardiac Surgery, University Hospital Zurich and University of Zurich, Zurich, Switzerland; Department of Infectious Diseases and Hospital Epidemiology, University Hospital Zurich and University of Zurich, Zurich, Switzerland; Infectious Diseases Service, Lausanne University Hospital and University of Lausanne, Lausanne, Switzerland; Department of Infectious Diseases and Hospital Epidemiology, University Hospital Zurich and University of Zurich, Zurich, Switzerland

**Keywords:** infective endocarditis, Duke criteria, echocardiography, ^18^F-FDG PET/CT, prosthetic valve

## Abstract

**Background:**

Since publication of Duke criteria for infective endocarditis (IE) diagnosis, several modifications have been proposed. We aimed to evaluate the diagnostic performance of the Duke-ISCVID (International Society of Cardiovascular Infectious Diseases) 2023 criteria compared to prior versions from 2000 (Duke-Li 2000) and 2015 (Duke-ESC [European Society for Cardiology] 2015).

**Methods:**

This study was conducted at 2 university hospitals between 2014 and 2022 among patients with suspected IE. A case was classified as IE (final IE diagnosis) by the Endocarditis Team. Sensitivity for each version of the Duke criteria was calculated among patients with confirmed IE based on pathological, surgical, and microbiological data. Specificity for each version of the Duke criteria was calculated among patients with suspected IE for whom IE diagnosis was ruled out.

**Results:**

In total, 2132 episodes with suspected IE were included, of which 1101 (52%) had final IE diagnosis. Definite IE by pathologic criteria was found in 285 (13%), 285 (13%), and 345 (16%) patients using the Duke-Li 2000, Duke-ESC 2015, or the Duke-ISCVID 2023 criteria, respectively. IE was excluded by histopathology in 25 (1%) patients. The Duke-ISCVID 2023 clinical criteria showed a higher sensitivity (84%) compared to previous versions (70%). However, specificity of the new clinical criteria was lower (60%) compared to previous versions (74%).

**Conclusions:**

The Duke-ISCVID 2023 criteria led to an increase in sensitivity compared to previous versions. Further studies are needed to evaluate items that could increase sensitivity by reducing the number of IE patients misclassified as possible, but without having detrimental effect on specificity of Duke criteria.

## Teaser

The new Duke-ISCVID (International Society of Cardiovascular Infectious Diseases) 2023 clinical criteria showed higher sensitivity (84%) compared to the previous versions (70%). The specificity of the Duke-ISCVID 2023 clinical criteria was lower (60%) compared to previous versions (74%).

## Graphical Abstract

**Graphical Abstract:**
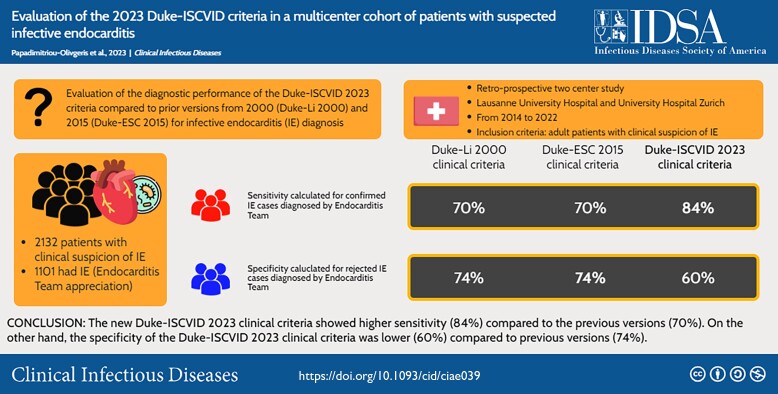
This graphical abstract is also available at Tidbit: https://tidbitapp.io/tidbits/evaluation-of-the-2023-duke-iscvid-criteria-in-a-multicenter-cohort-of-patients-with-suspected-infective-endocarditis

**(See the Invited Commentary by Chambers et al. on pages 964–7.)**

Advances in microbiology and imaging have improved our capacity to diagnose infective endocarditis (IE) but have not changed its epidemiology [1]. Early identification of IE is crucial to improve outcomes [1–4]. In 1994, the Duke criteria were introduced to standardize the diagnosis of IE for research purposes. These criteria were revised in 2000 and have since been widely used in clinical practice, with a sensitivity of approximately 80% [5–8]. However, they have been found to be less effective in cases of prosthetic valve IE or cardiac implantable electronic device (CIED)-lead IE, where echocardiography can be inconclusive [9].

In response, the 2015 European Society of Cardiology (ESC) Clinical Practice Guidelines proposed modifications to the Duke criteria, including the addition of cardiac CT and ^18^F-Fluorodeoxyglucose Positron Emission Tomography/Computed Tomography (^18^F-FDG PET/CT) for prosthetic valve IE, as well as the omission of “significant new valvular regurgitation on echocardiography as compared to previous imaging” from the major imaging criterion [4]. Although the ESC 2015 Duke criteria, significantly improved diagnostic accuracy, especially for prosthetic valve IE, they failed to address improvements of microbiologic diagnostics.

Therefore, the International Society for Cardiovascular Infectious Diseases (ISCVID) proposed a thorough revision of the Duke criteria in 2023. The 2023 Duke-ISCVID Criteria for Infective Endocarditis include adaptations to microorganisms that commonly cause IE and now also consider some pathogens as typical only in the presence of intracardiac prostheses. Moreover, the microbiology criteria now incorporate enzyme immunoassay for *Bartonella* and *Brucella* species, polymerase chain reaction (PCR), amplicon/metagenomic sequencing, and in situ hybridization. In addition, imaging criteria were adapted by adding cardiac CT and ^18^F-FDG PET/CT for the diagnosis of both native and prosthetic valve endocarditis, and ^18^F-FDG PET/CT for CIED pocket and lead infection. Furthermore, the 2023 Duke-ISCVID criteria also include intraoperative evidence of IE as a new surgical major criterion. Transcatheter aortic valve implantation (TAVI) and endovascular CIED have been added to cardiac predisposing criteria, and cerebral or splenic abscess to vascular phenomena [10].

The aim of our study was to evaluate the diagnostic performance of the new 2023 Duke-ISCVID criteria for IE compared to the 2 previous versions (Duke-Li 2000 and Duke-ESC 2015 criteria) [4, 8] in a multicenter study of patients with suspicion of IE.

## METHODS

This multicenter study took place at the Lausanne University Hospital (CHUV) and the University Hospital Zurich (USZ). The CHUV cohort was divided in 2 parts: a retrospective cohort of IE cases from 2014 to2017 and a prospective cohort of cases with suspected IE from 2018 to 2022. The USZ cohort was also divided in 2 parts: a retrospective cohort of IE from 2014 to2017 and a prospective cohort of IE from 2018 to 2022. The Ethics Committees of the Canton of Vaud and Canton of Zurich approved the study (CER-VD 2017-02137, KEK-2014-0461; BASEC 2017-01140).

Adult patients (≥18 years old) who were hospitalized with IE were included. In addition, for the period 2018 to 2022 in CHUV patients with suspected IE (patients who had blood cultures drawn and an echocardiography performed specifically for the research of IE) were included. Exclusion criterion for the retrospective cohorts was refusal to use their data and for the prospective cohorts the absence of written consent.

Demographic, clinical (comorbidities, cardiac predisposing factors, fever, vascular or immunologic phenomena), and microbiological data were collected from patient's electronic health charts. A case was classified as IE (final IE diagnosis) by the Endocarditis Team from each center at day 60, based on clinical, microbiological, imaging, surgical data, or autopsy results. Cases were classified as rejected, possible or definite IE based on clinical criteria alone or by a composite of clinical and pathological criteria stratified by the three versions of Duke criteria (Duke-Li 2000 [8], Duke-ESC 2015 [4], and Duke-ISCVID 2023 [10]). Infection site and alternative firm diagnosis were defined by the infectious diseases specialist responsible of the case based on clinical, radiological, microbiological, and operative findings.

### Management of IE

According to internal guidelines of both hospitals, an infectious diseases consultation with a thorough physical examination was performed on a mandatory basis for all patients with suspected IE. ^18^F-FDG PET/CT or cardiac CT was proposed in selected cases by the endocarditis team. Thoraco-abdominal and cerebral imaging were performed in all patients with clinical suspicion of embolic events. Imaging in asymptomatic patients was left at the discretion of the treating physician and infectious diseases consultant.

### Statistical Analysis

For data analysis, SPSS version 26.0 (SPSS, Chicago, Illinois, USA) software was used. Group differences were investigated using the Mann-Whitney *U* test for continuous variables and the χ^22^ or Fisher exact test for categorical variables. As suggested by the 2023 Duke-ISCVID criteria, we calculated for each version of the clinical Duke criteria, the sensitivity among patients with confirmed IE and specificity among patients with suspected IE for whom IE diagnosis was ruled out [10]. More specifically, sensitivity was calculated among patients with confirmed IE based on: (1) positive valve culture; (2) positive molecular assay of valve tissue with the same pathogen as isolated from blood cultures; (3) positive valve histopathology; (4) positive CIED-lead culture with the same pathogen as isolated from blood cultures in patients whose leads were extracted without contact with infected pocket site (surgical extraction, transvenous lead extraction without infected pocket site); (5) and positive macroscopic evidence (surgery or autopsy) of IE. Specificity for each version of the clinical Duke criteria was calculated among patients with suspected IE for whom IE diagnosis was ruled out: (1) negative valve histopathology, (2) non-infectious diagnosis, (3) infected patients cured by short antibiotic course (<14 days). All tests were 2-tailed, and *P* < .05 was considered statistically significant.

## RESULTS

**Figure 1.**
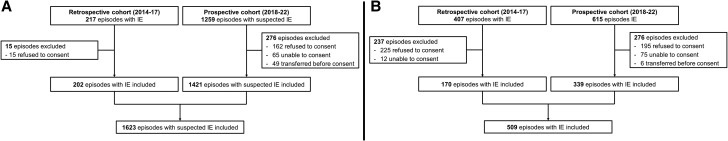
Flowchart of included patients in (*A*) Lausanne University Hospital and (*B*) in University Hospital Zurich. Abbreviation: IE, infective endocarditis.

Among the 2498 episodes with suspected IE, 2132 were included (Figure 1); 1623 (76%) in CHUV and 509 (24%) in USZ. Thereof, 1101 (52%) had final diagnosis of IE (592 in CHUV and 509 in USZ); 710 (64%) native valve; 252; (23%) prosthetic valve; 160 (15%) CIED-lead and 5 (0.5%) other intracardiac structure). Among the 1031 patients without IE, other infectious diagnoses predominated (855; 83%) (Supplementary Table 1).

Patients’ characteristics with emphasis on difference between different versions of Duke criteria are shown in Table 1. Definite IE by clinical criteria was found in 692 (32%), 695 (33%), and 871 (41%) patients using the Duke-Li 2000, Duke-ESC 2015, or the Duke-ISCVID 2023 criteria, respectively. Definite IE by pathologic criteria was found in 285 (13%), 285 (13%), and 345 (16%) patients using the Duke-Li 2000, Duke-ESC 2015 or the Duke-ISCVID 2023 criteria, respectively. IE was excluded by histopathology or macroscopic evidence at surgery or autopsy with antibiotic treatment for <4 days in 25 (1%) patients. Supplementary Figure 1 shows the classification of episodes by different versions of Duke criteria based on clinical criteria prior or after application of pathological confirmation and pathological rejection criteria.

**Table 1.** Comparison of Episodes With or Without Final Infective Endocarditis Diagnosis Among 2132 Patients With Suspected Infective Endocarditis

	No Infective Endocarditis (n = 1031)		Infective Endocarditis (n = 1101)		*P* Value
Demographics					
Male sex, n (%)	695	(67)	828	(75)	<.001
Age (median years, IQR)	67	(55–77)	65	(50–75)	.01
Charlson comorbidity index, (median, IQR)	5	(2–7)	4	(2–6)	<.001
Cardiac predisposing factors					
IV drug use, n (%)	48	(5)	222	(10)	<.001
Rheumatic heart disease/Hypertrophic cardiomyopathy, n (%)	2	(.2)	16	(2)	.001
Congenital disease, n (%)	24	(2)	207	(19)	<.001
Prosthetic valve, n (%)	80	(8)	302	(27)	<.001
*Prior endocarditis*, n (%)	34	(3)	140	(13)	<.001
*Moderate or severe valve regurgitation/stenosis*, n (%)	101	(10)	251	(23)	<.001
*Cardiac implantable electronic devices*, n (%)	100	(10)	353	(32)	<.001
*Transcatheter aortic valve replacement*, n (%)	9	(0.9)	46	(4)	<.001
Minor predisposition criterion (Duke Li 2000), n (%)	150	(15)	541	(49)	<.001
Minor predisposition criterion (ESC 2015),	159	(15)	565	(51)	<.001
Minor predisposition criterion (ISCVID 2023), n (%)	300	(29)	821	(75)	<.001
Microbiological data					
Bacteremia/fungemia, n (%)	648	(63)	990	(90)	<.001
*S. aureus*, n (%)	293	(28)	417	(38)	<.001
Coagulase-negative staphylococci, n (%)	59	(6)	67	(6)	.783
*S. lugdunensis*, n (%)	9	(0.9)	13	(1)	.526
*Coagulase-negative staphylococci in the presence of intracardiac prosthetic material*, n (%)	30	(3)	34	(3)	.920
*Coagulase-negative staphylococci isolated from 3 or more separate blood culture sets*, n (%)	17	(2)	42	(4)	.281
*Streptococcus* spp.	115	(11)	290	(26)	<.001
*S. gallolyticus*, n (%)	9	(0.9)	47	(4)	<.001
Viridans streptococci, n (%)	65	(6)	183	(17)	<.001
*Group B, C, or G streptococci*, n (%)	21	(2)	49	(5)	.002
S. pneumoniae *or *S. pyogenes* isolated from 3 or more separate blood culture sets*, n (%)	2	(0.2)	4	(0.4)	.688
*Enterococcus* spp., n (%)	80	(8)	130	(12)	.002
*Community-acquired enterococci without known primary focus*, n (%)	17	(2)	103	(9)	<.001
*E. faecalis*, n (%)	54	(5)	114	(10)	<.001
*Enterococci other than* E. faecalis *isolated from 3 or more separate blood culture sets*, n (%)	8	(1)	14	(1)	.290
Gram-positive (other than staphylococci, streptococci, and enterococci), n (%)	26	(3)	32	(3)	.597
*Abiotrophia* spp., n (%)	1	(0.1)	5	(0.5)	.220
*Granulicatella* spp., n (%)	3	(0.3)	2	(0.2)	.678
*Gemella* spp., n (%)	0	(0)	1	(0.1)	1.000
*Cutibacterium acnes* in the presence of intracardiac prosthetic material, n (%)	1	(0.1)	11	(1)	.007
*Corynebacterium* spp*. in the presence of intracardiac prosthetic material*, n (%)	1	(0.1)	5	(0.5)	.220
*Gram-positive (other than staphylococci, streptococci, and enterococci) isolated from 3 or more separate blood culture sets*, n (%)	7	(0.7)	17	(2)	.066
HACEK, n (%)	3	(0.3)	26	(2)	<.001
Gram-negative (other than HACEK), n (%)	107	(10)	26	(2)	<.001
Pseudomonas aeruginosa *in the presence of intracardiac prosthetic material*, n (%)	4	(0.4)	1	(0.1)	.204
Serratia marcescens *in the presence of intracardiac prosthetic material*, n (%)	1	(0.1)	0	(0)	.484
*Other gram-negative (other than HACEK) isolated from 3 or more separate blood culture sets*, n (%)	31	(3)	14	(1)	.006
Fungi, n (%)	37	(4)	10	(0.9)	<.001
Candida spp. *in the presence of intracardiac prosthetic material*, n (%)	5	(0.5)	6	(0.5)	1.000
*Fungi isolated from 3 or more separate blood culture sets*, n (%)	12	(1)	4	(.4)	.043
*Microorganisms that occasionally or rarely cause IE isolated from 3 or more separate blood culture sets*, n (%)	73	(7)	50	(5)	.012
Polymicrobial bacteremia, n (%)	76	(7)	23	(2)	<.001
Culture negative investigations, n (%)	…	…	…	…	
*Coxiella burnetii* antiphase I IgG titer ≥1:800, n (%)	2	(0.2)	8	(0.7)	.110
*Positive blood PCR for* C. burnetii, n (%)	1	(0.1)	1	(0.1)	1.000
Bartonella henselae *or *B. quintana* IgG titer ≥1:800,* n (%)	7	(.7)	4	(0.4)	.373
*Positive culture for an organism consistent with IE from a sterile body site other than cardiac tissue, cardiac prosthesis, or embolus*, n (%)	227	(22)	91	(8)	<.001
Major microbiological criterion (Li 2000), n (%)	275	(27)	734	(67)	<.001
Major microbiological criterion (ESC 2015), n (%)	275	(27)	734	(67)	<.001
Major microbiological criterion (ISCVID 2023), n (%)	412	(40)	907	(82)	<.001
Minor microbiological criterion (Li 2000), n (%)	165	(16)	72	(7)	<.001
Minor microbiological criterion (ESC 2015), n (%)	165	(16)	72	(7)	<.001
Minor microbiological criterion (ISCVID 2023), n (%)	188	(18)	61	(6)	<.001
Imaging data					
Positive echocardiography (either TTE or TOE) for vegetation, perforation, abscess, aneurysm, pseudoaneurysm, fistula, n (%)	23	(2)	833	(76)	<.001
*Abnormal metabolic activity in ^18^F-FDG PET/CT in native or prosthetic valve or CIED lead*, n (%)	5	(1)	109	(10)	<.001
*Abnormal metabolic activity in ^18^F-FDG PET/CT in prosthetic valve*, n (%)	3	(0.3)	70	(6)	<.001
*Positive cardiac-CT for vegetation, perforation, abscess, aneurysm, pseudoaneurysm, fistula*, n (%)	2	(0.2)	40	(4)	<.001
*Significant new valvular regurgitation on echocardiography as compared to previous imaging*, n (%)	21	(2)	207	(19)	<.001
Major imaging criterion (Li 2000), n (%)	41	(4)	852	(77)	<.001
Major imaging criterion (ESC 2015), n (%)	25	(2)	877	(80)	<.001
Major imaging criterion (ISCVID 2023), n (%)	44	(4)	913	(83)	<.001
Manifestations	…	…	…	…	
Minor fever criterion (all versions), n (%)	788	(76)	860	(78)	.379
New heart murmur, n (%)	160	(16)	410	(37)	<.001
Vascular phenomena (major arterial emboli, septic pulmonary infarcts, mycotic aneurysm, intracranial hemorrhage, conjunctival hemorrhages, and Janeway's lesions), n (%)	156	(15)	590	(54)	<.001
*Cerebral abscess*, n (%)	8	(0.8)	3	(0.3)	.134
*Splenic abscess*, n (%)	0	(0)	1	(0)	1.000
Minor vascular criterion (Li 2000), n (%)	156	(15)	590	(54)	<.001
Minor vascular criterion (ESC 2015), n (%)	156	(15)	590	(54)	<.001
Minor vascular criterion (ISCVID 2023), n (%)	161	(16)	591	(54)	<.001
Minor immunologic criterion (all versions), n (%)	17	(2)	144	(13)	<.001
Data on surgery/CIED extraction/histopathology, n (%)					
Valve surgery performed, n (%)	16	(2)	448	(41)	<.001
Major surgery criterion (ISCVID 2023), n (%)	0	(0)	12	(1)	.001
CIED extraction (among 453 patients with CIED), n (%)	17	(17)	109	(31)	<.001
*Positive CIED-lead culture (without contact with infected pocket site)*, n (%)	0	(0)	46	(13)	<.001
*Macroscopic evidence of IE by inspection (surgery/autopsy),* n (%)	5	(.5)	297	(27)	<.001
Autopsy performed, n (%)	14	(1)	17	(2)	.857
Histopathology compatible for IE or positive culture or of vegetation, abscess, or embolized lesion, n (%)	0	(0)	285	(26)	<.001
*Positive nucleic acid-based tests*, n (%)	0	(0)	42	(4)	<.001
Duke pathological criterion (Li 2000), n (%)	0	(0)	285	(26)	<.001
Duke pathological criterion (ESC 2015), n (%)	0	(0)	285	(26)	<.001
Duke pathological criterion (ISCVID 2023), n (%)	0	(0)	345	(31)	<.001
Exclusion pathological criterion (all versions), n (%)	25	(2)	0	(0)	<.001
Outcome					
Lack of recurrence despite antibiotic therapy for <4 d (absence of death within 7d), n (%)	167	(16)	0	(0)	<.001
Lack of recurrence despite antibiotic therapy for <4 d among cases with infectious diagnoses (absence of death within 7d), n (%)	33	(4)	0	(0)	<.001
Classifications					
Classification according to Duke Li 2000 clinical criteria					
Rejected, n (%)	706	(69)	73	(7)	
Possible, n (%)	316	(31)	345	(31)	
Definite, n (%)	9	(1)	683	(62)	<.001
Classification according to Duke-ESC 2015 clinical criteria					
Rejected, n (%)	715	(69)	59	(5)	
Possible, n (%)	312	(30)	351	(32)	
Definite, n (%)	4	(0.4)	691	(63)	<.001
Classification according to Duke-ISCVID 2023 clinical criteria					
Rejected, n (%)	561	(54)	14	(1)	
Possible, n (%)	453	(44)	233	(21)	
Definite, n (%)	17	(2)	854	(78)	<.001

In *italics* appear the characteristics that differ between the different versions of the Duke criteria.

TTE, TOE, 18F-FDG PET/CT, and cardiac CT were performed in 2037 (96%), 1237 (58%), 396 (19%), and 77 (4%) patients, respectively. In CHUV, thoracoabdominal and cerebral imaging for the research of embolic events were performed in 1169 (72%) and 641 (39%) patients, respectively. Valve surgery, CIED extraction, and autopsy were performed in 464 (22%) patients 31 (1%) and 126 (out of 453 patients with CIED; 28%) patients, respectively.

Abbreviations: ^18^F-FDG PET/CT, ^18^F-fluorodeoxyglucose positron emission tomography/computed tomography; CIED, cardiac implantable electronic devices; ESC, European Society of Cardiology; HACEK, *Haemophilus* spp., *Aggregatibacter* spp., *Cardiobacterium hominis*, *Eikenella corrodens*, *Kingella kingae*; IE, infective endocarditis; IgG, immunoglobulin G; IQR, interquartile range; ISCVID, International Society of Cardiovascular Infectious Diseases.

The performance of the different versions of the Duke clinical criteria stratified by different groups is depicted in Table 2. The 2023 Duke-ISCVID criteria for IE showed higher sensitivity (84%) as compared to the previous versions (70%). However, the specificity of the clinical criteria showed lower specificity (60%) as compared to previous versions (74%). Non-IE cases misclassified as definite IE by any version of Duke clinical criteria are shown in Supplementary Table 2, whereas IE cases misclassified as rejected IE by any version of Duke clinical criteria are shown in Supplementary Table 3.

**Table 2.** Performance of Different Versions of the Duke Clinical Criteria Among Different Groups

Patient Group	Type of Calculation	Number of Patients n	Duke-Li 2000 Criteria % 95% CI %	Duke-ESC 2015 Criteria % 95% CI %	Duke-ISCVID 2023 Criteria % 95% CI %
Confirmed IE patients	Sensitivity^a^	443	70 (65–74)	70 (65–74)	84 (81–87)
Prosthetic valve	Sensitivity^a^	113	66 (57–75)	69 (60–77)	84 (76–91)
Native valve	Sensitivity^a^	312	74 (57–75)	73 (68–78)	88 (84–92)
Rejected IE cases	Specificity^b^	731	74 (70–76)	74 (71–77)	60 (56–63)

Abbreviations: CI, confidence interval; ESC, European Society for Cardiology; IE, infective endocarditis; ISCVID, International Society of Cardiovascular Infectious Diseases.

^a^Definite IE cases by Duke clinical criteria being diagnosed as IE by the Endocarditis Team.

^b^Rejected cases by Duke clinical criteria being diagnosed as non-IE by the Endocarditis Team.

## DISCUSSION

The proposed changes to the Duke criteria by the ISCVID 2023 led to an increase in sensitivity as compared to previous versions, but a decrease in specificity.

In earlier studies, the sensitivity of the initial Duke criteria and the proposed changes by Li et al in 2000 was approximately 80%–88% [5–8]. However, in the subgroup of patients with prosthetic valve IE, the sensitivity was lower (50%–57%) [7, 11]. The addition of ^18^F-FDG PET/CT in the ESC 2015 criteria increased sensitivity in that subgroup to 84% [11]. The 2023 Duke-ISCVID Criteria for IE expanded the utility of the ^18^F-FDG PET/CT in patients with native valves and CIED-lead IE [10]. A previous meta-analysis showed that sensitivity of ^18^F-FDG PET/CT in patients with prosthetic valve IE and CIED-lead IE was 86% and 72%, respectively, both higher than for native valve IE (31%) [12]. Therefore, ^18^F-FDG PET/CT could be an important addition in the diagnostic work-up of patients with suspected IE and prosthetic valve or CIED, for which echocardiography is known to inconclusive or unspecific in many patients [9]. Although sensitivity in native valve IE was low, ^18^F-FDG PET/CT inclusion in the diagnostic algorithm in selected patients whose IE diagnosis is not yet proven by echocardiography, despite a high clinical suspicion, could be useful.

One significant change in the Duke-ISCVID 2023 was the inclusion of the macroscopic findings during surgery/autopsy as a major criterion [10]. In our study, among patients with final IE diagnosis, macroscopic findings suggestive of IE were found in 297 (64%) of 465 patients that had valve surgery or autopsy. However, only 12 (1%) patients fulfilled the new major criterion, since for the remainder, the major imaging criterion was already established. Although the impact of this new major criterion was minimal in the present study due to the extensive inclusion in our diagnostic algorithm of ^18^F-FDG PET/CT and cardiac CT, its significance could be essential in centers where these imaging modalities are not widely available [13]. Intracardiac structures can be visualized visually during surgery and by cardiac imaging. Thus, one could also unify these 2 criteria.

There were also major changes to the microbiologic criteria [10]. First, the list of pathogens that commonly cause IE was updated with the incorporation of infrequent pathogens such as *Staphylococcus lugdunensis, Streptococcus agalactiae, Streptococcus dysgalactiae, Granulicatella* spp.*, Abiotrophia* spp*.,* and *Gemella* spp. However, the most important change was the substitution of “community-acquired enterococci, in the absence of a primary focus” with “*E. faecalis.*” The latter change was spearheaded by the publication from Dahl et al [14], which showed an increased prevalence of IE in patients with a known source compared to previous studies [15, 16]. In the present study, the discriminatory capacity of the former definition was better than the latter. With the Duke-ISCVID 2023 definition, the major criterion was established in 114 cases of final IE diagnosis (compared to 103 with the former), although it falsely attributed the major criterion in 54 patients without IE compared to only 17 with the former. Concerning the mode of acquisition, as shown in previous studies, enterococcal IE was rare in nosocomial bacteremia (∼3%) [14–16]. Thus, to avoid unnecessary echocardiograms, especially in patients at low risk, such as those with nosocomial bacteremia, the criterion could be changed to “non-nosocomial-acquired enterococci.”

The second change in the list of pathogens that commonly cause IE was the addition of the subset of patients with prosthetic intracardiac material and at least 2 positive blood culture sets for different pathogens [10]. Due to the low occurrence of IE by these pathogens (only 6% of IE cases in our study), their incorporation in the major criterion will slightly increase sensitivity, but it will certainly lead to a decrease in specificity (4% fulfilled the criterion in the non-IE group). A major implication of this change could be the increase of the number of unnecessary echocardiograms, because Duke criteria are not only used for research purposes but also employed in clinical practice. In our centers, all patients with suspected IE are evaluated by infectious diseases physicians. However, this may not be the case in many non-university centers, which base the use of echocardiography on the Duke criteria. For the same reason, the inclusion of the subset of pathogens that occasionally or rarely cause IE isolated from at least 3 separate blood culture sets could hinder specificity without adding significantly in sensitivity.

The 2 previous versions of Duke criteria, included as rejection criterion reading as “resolution of symptoms within 4 days of introduction of antibiotic therapy,” which was arbitrary excluding many patients with final IE diagnosis. In the Duke-ISCVID 2023, the aforementioned criterion was replaced by “Lack of recurrence despite antibiotic therapy for less than 4 days” [4, 8, 10]. This criterion led to rejection of only 4% of patients without IE but other infectious diagnosis; thus its applicability did not substantially aid IE diagnosis, because only a minority of patients with infection could be treated with 4 days or less. A possible adaptation of the new criterion could be “Lack of recurrence despite antibiotic therapy for <14 days if treated by monotherapy or 7 if treated by combination treatment” because a monotherapy of 14 days might not be sufficient to treat an IE.

The present study has several limitations. Although the study had a moderate number of patients, it was not large enough to evaluate changes in less frequent events such as cerebral or splenic abscess and bacteremia by rare pathogens. Nonetheless, the study included patients with suspected and ultimately rejected IE, unlike previous studies that primarily focused on patients with possible or definite IE [5–8, 11, 17]. Another limitation is that both centers are based in Switzerland, and thus, the epidemiology could be different in other regions. This was shown in a previous study from South Africa with a higher incidence of intracellular pathogens such as *Bartonella* spp [18]. Furthermore, all patients with suspected IE were examined by an infectious diseases specialist, leading to improved clinical detection of vascular or immunologic phenomena not previously described by treating physicians and to a more systematic prescription of additional imaging studies for embolic event detection [19, 20]. This could explain the high percentage of embolic events found in the present study (55%) compared to previous ones (∼35%) [21–23]. Therefore, the results of the present study cannot be extrapolated to centers in which patients with suspected IE are not evaluated by infectious diseases specialists.

In conclusion, the 2023 Duke-ISCVID criteria for IE led to an increase in sensitivity compared to previous versions. Furthermore, our study demonstrated the diagnostic value of ^18^F-FDG PET/CT and cardiac CT in patients whose IE diagnosis is not yet proven by echocardiography, despite a high clinical suspicion. Further studies are needed to evaluate items that could increase sensitivity by reducing the number of IE patients misclassified as possible, but without having detrimental effect on specificity of the Duke criteria, which are not primarily intended for clinical practice, but for research application.

## Supplementary Data

Supplementary materials are available at *Clinical Infectious Diseases* online. Consisting of data provided by the authors to benefit the reader, the posted materials are not copyedited and are the sole responsibility of the authors, so questions or comments should be addressed to the corresponding author.

## Supplementary Material

ciae039_Supplementary_Data

## Notes

***Author contributions*.** B. H. and M. P. O. conceived the idea. M. P. O., P. M., M. F., G. T., P. T., M. K., M. V. H., R. B., J. E., B. G., and B. H. collected the patients’ data. B. H. and M. P. O. supervised the project. M. P. O. and B. H. performed the analysis. All authors interpreted the results. M. P. O. wrote the first draft of the manuscript. All authors contributed to manuscript revision and read and approved the submitted version.

***Financial support*.** This work was supported by the Swiss National Science Foundation (SNSF) grant number 32003B_219351/1 (to B. H. and M. P. O.) and the Clinical Research Priority Program (CRPP) of the University of Zurich for the CRPP Precision medicine for bacterial infections (to B. H.). The funders had no role in study design, data collection and analysis, decision to publish, or preparation of the manuscript.
