# Supplementary material for: Evaluation of the 2023 Duke-International Society of Cardiovascular Infectious Diseases Criteria in a Multicenter Cohort of Patients With Suspected Infective Endocarditis
Source: Clin Infect Dis. 2024 Feb 8;78(4):949–55. doi: 10.1093/cid/ciae039 (PMC11006096; doi:10.1093/cid/ciae039)

Supplementary Table 1 Characteristics of patients without final infective endocarditis diagnosis

	No infective endocarditis (n=1031)	
**Final diagnosis**		
Other type of infection, n (%)	855	(83)
Stroke, n (%)	25	(2)
Malignancy-related fever, n (%)	21	(2)
Fever of unknown origin, n (%)	36	(4)
Autoimmune disease, n (%)	23	(2)
Non-infective endocarditis, n (%)	12	(1)
Other diagnoses, n (%)	59	(6)

Supplementary Table 2. Misclassification of non-infective endocarditis cases as definite infective endocarditis by clinical criteria (by any version of Duke criteria)

	Microbiologic criterion (positive bcs)	Imaging criterion	Predisposition	Fever	Vascular phenomena	Immunologic phenomena	Valve surgery	Pathological exclusion criterion	Final diagnosis
1	Bacteremia *S. aureus* (2 bcs)	Significant new valvular regurgitation		Y			N	N	Catheter-related bacteremia
2	Bacteremia *S. mitis* (2 bcs)	Significant new valvular regurgitation	Moderate valve stenosis	Y			Y	Y	Oral abscess
3	Bacteremia *C. hominis* (1 bcs)	Significant new valvular regurgitation	Prosthetic valve, prior IE	Y			N	N	Auto-immune diseases (translocation)
4	Bacteremia *S. anginosus* (2 bcs)		Prosthetic valve	Y	Pulmonary septic emboli	Roth's spot	N	N	Catheter-related bacteremia with septic thrombophlebitis
5		Vegetation, significant new valvular regurgitation	Prosthetic valve	Y	Cerebral emboli		Y	Y	Marantic IE
6	Bacteremia *S. aureus* (2 bcs)		IV drug use, prosthetic valve	Y	Peripheral mycotic aneurysm		N	N	Contiguous mycotic aneurysm (IV drug use)
7		Significant new valvular regurgitation	Prosthetic valve	Y	Cerebral emboli		Y	Y	Marantic IE
8		Significant new valvular regurgitation		Y	Cerebral emboli	Positive rheumatoid factor	N	N	Pneumonia
9	Bacteremia *S. pneumoniae* (2 bcs)	Significant new valvular regurgitation	Bicuspid aortic valve	Y	Cerebral emboli		N	N	Pneumonia
10	*B. henselae* IgG titer ≥1:800	Significant new valvular regurgitation		N			N	N	Paradoxal reaction to tuberculosis treatment

11	Bacteremia (nosocomial) *E. faecalis* (2 bcs)	Abnormal metabolic activity (^{18}F-FDG PET/CT) of native valve		N			N	N	Catheter-related bacteremia
12	Bacteremia *S. epidermidis* (2 bcs)		Moderate valve regurgitation, CIED	N	Pulmonary septic emboli		N	N	Catheter-related bacteremia with septic thrombophlebitis
13	Bacteremia *L. garvieae* (3 bcs)		Prosthetic valve	Y	Cerebral emboli		N	N	Spondylodiscitis (cerebral emboli due to atrial fibrillation)
14	Bacteremia *S. epidermidis* (3 bcs)		Prior IE	Y		Roth's spot	N	N	Catheter-related bacteremia with septic thrombophlebitis
15	Bacteremia *S. aureus* (2 bcs)		IV drug use	Y	Pulmonary septic emboli		N	N	Cellulitis with septic thrombophlebitis
16	Bacteremia *S. aureus* (2 bcs)		CIED	Y	Pulmonary septic emboli		N	N	Catheter-related bacteremia with septic thrombophlebitis
17	Candidemia *C. tropicalis* (3 bcs)			Y	Ocular emboli	Roth's spot	N	N	Catheter-related urinary tract infection

^{18}F-FDG PET/CT: ^{18}F-Fluorodeoxyglucose Positron Emission Tomography/Computed Tomography; bcs: blood culture set; CIED: cardiac implantable electronic device; IE: infective endocarditis; N: no; Y: yes

Supplementary table 3. Misclassification of infective endocarditis cases as rejected by clinical criteria (by any version of Duke criteria)

	**Microbiologic criterion (positive bcs)**	**Imaging criterion**	**Predisposition**	**Fever**	**Vascular phenomena**	**Immunologic phenomena**	**Valve surgery**	**Surgical criterion**	**Pathological criterion**	**Other information**
1	Bacteremia *S. epidermidis* (2 bcs)	Abnormal metabolic activity (^{18}F-FDG PET/CT) of native valve	Prior IE (*S.epidermidis*)	N			N	N	N	
2	On antibiotic treatment	CIED-lead lesion (degenerative)	CIED	N			N	N	N	Positive CIED-lead culture
3	On antibiotic treatment	CIED-lead lesion (degenerative)	CIED	N			N	N	N	Positive CIED-lead culture
4	On antibiotic treatment	CIED-lead lesion (degenerative)	CIED	N			N	N	N	Positive CIED-lead culture
5	On antibiotic treatment	CIED-lead lesion (degenerative)	CIED	N			N	N	N	Positive CIED-lead culture
6	Bacteremia *S. epidermidis* (2 bcs)	Abnormal metabolic activity (^{18}F-FDG PET/CT) of CIED cable	CIED	Y			N	N	N	Positive CIED-lead culture
7	Bacteremia *S. agalactiae* (2 bcs)	Abnormal metabolic activity (^{18}F-FDG PET/CT) of native valve		Y			N	N	N	
8	Bacteremia *B. pumilus* (2 bcs)	Abnormal metabolic activity (^{18}F-FDG PET/CT) of TAVI	TAVI	Y			N	N	N	
9	Bacteremia (persistent) *S. epidermidis* (2 bcs)		TAVI	Y			N	N	N	
10	Bacteremia (persistent) *S. epidermidis* (2		TAVI	Y			N	N	N	

	bcs)							
11	Bacteremia *S. agalactiae* (2 bcs)	Small mobile element (degenerative)	TAVI	Y	N	N	N	
12	Bacteremia (persistent) *S. epidermidis* (2 bcs)		Prosthetic valve, CIED, prior IE (*S. epidermidis*)	Y	Y	Y	Y	Positive CIED-lead culture
13	Bacteremia *S. dysgalactiae* (2 bcs)	Small mobile element (degenerative)		Y	N	N	N	
14	Bacteremia *S. agalactiae* (3 bcs)	Significant new valvular regurgitation		Y	N	N	N	
15	On antibiotic treatment		Bicuspid aortic valve	N	Y	Y	Y	
16	Bacteremia *C. fetus* (2 bcs)	Small mobile element (degenerative), abnormal metabolic activity (^{18}F-FDG PET/CT) of native valve		N	N	N	N	
17	On antibiotic treatment	CIED-cable lesion (degenerative)	Prosthetic valve, CIED	N	N	N	N	Positive CIED-lead culture
18	On antibiotic treatment	CIED-lead vegetation	CIED	N	N	N	N	
19	On antibiotic treatment	CIED-lead vegetation, valve vegetation	CIED	N	N	N	N	Positive CIED-lead culture
20	Bacteremia *S. aureus* (2 bcs)		CIED	N	N	N	N	Positive CIED-lead culture
21	Bacteremia *S. epidermidis* (2 bcs)	CIED-lead vegetation	CIED	N	N	N	N	Positive CIED-lead culture

22	Bacteremia (community-acquired) *E. faecium* (1 bcs)	CIED-lead vegetation, abnormal metabolic activity (^{18}F-FDG PET/CT) of CIED cable	CIED	N		N	N	N	Positive CIED-lead culture
23	On antibiotic treatment	Significant new valvular regurgitation		Y		Y	Y	Y	
24	On antibiotic treatment	CIED-lead vegetation	CIED	N		N	N	N	Positive CIED-lead culture
25	On antibiotic treatment	Valve vegetation	Prior IE	N		N	N	N	
26	Bacteremia (community-acquired) *E. faecalis* (2 bcs)	Significant new valvular regurgitation	Severe valve stenosis	N		N	N	N	
27	Bacteremia *S. lugdunensis* (2 bcs)	Abnormal metabolic activity (^{18}F-FDG PET/CT) of prosthetic valve	Prosthetic valve	Y		Y	Y	N	
28	Candidemia *C. glabrata* (3 bcs)	Abnormal metabolic activity (^{18}F-FDG PET/CT) of prosthetic valve	Prosthetic valve	Y		N	N	N	
29	*B. henselae* IgG titer ≥1:800	Abnormal metabolic activity (^{18}F-FDG PET/CT) of prosthetic valve	Prosthetic valve	Y		N	N	N	
30	Bacteremia *P. aeruginosa* (2 bcs)	Thickening valve leaflet, abnormal metabolic activity (^{18}F-FDG	Moderate valve stenosis	Y		N	N	N	

		PET/CT) of native valve						
31	On antibiotic treatment	Abnormal metabolic activity (^{18}F-FDG PET/CT) of prosthetic valve	Prosthetic valve	Y		N	N	N
32	On antibiotic treatment	Small mobile element (degenerative), abnormal metabolic activity (^{18}F-FDG PET/CT) of native valve, abscess on cardiac CT		Y	Arterial emboli, cerebral emboli	N	N	N
33	On antibiotic treatment		Prosthetic valve	N		Y	Y	N
34	Bacteremia (persistent) *S. epidermidis* (2 bcs)	Small mobile element (degenerative)	Prosthetic valve	Y		N	N	N
35	Bacteremia (nosocomial) *E. faecalis* (2 bcs)	Thickening valve leaflet	TAVI, CIED	Y		N	N	N
36	Bacteremia *S. enteritidis* (2 bcs)	Abnormal metabolic activity (^{18}F-FDG PET/CT) of native valve		Y	Pulmonary septic emboli	N	N	N
37	On antibiotic treatment		Prior IE	Y	Cerebral emboli	N	N	N
38	Bacteremia *S. epidermidis* (2 bcs)		Severe valve regurgitation	Y	Pulmonary septic emboli, mycotic aneurysm	N	N	N

39	Bacteremia *S. pneumoniae* (2 bcs)	CIED-lead lesion (degenerative)	CIED	Y		N	N	N	Multiple septic arthritis
40	On antibiotic treatment	Thickening valve leaflet		Y		N	N	N	Multiple septic arthritis
41	Bacteremia *S. epidermidis* (2 bcs)	Thickening valve leaflet		N	Cerebral emboli	N	N	N	
42	Bacteremia *S. agalactiae* (2 bcs)		Bicuspid aortic valve	Y		Y	Y	Y	
43	Bacteremia (community-acquired) *E. faecalis* (2 bcs)	Small mobile element (degenerative)	Prosthetic valve	Y		N	N	N	
44	On antibiotic treatment	Thickening valve leaflet		Y	Mycotic aneurysm	N	N	N	Septic arthritis
45	Bacteremia *S. aureus* (1 bcs)	Thickening valve leaflet		Y		N	N	N	
46	Bacteremia (community-acquired) *E. faecalis* (1 bcs)	Thickening valve leaflet		N		N	N	N	Septic arthritis
47	Bacteremia *A. defectiva* (2 bcs)	Thickening valve leaflet	Prolapsus of mitral valve	Y		Y	Y	Y	
48	Bacteremia *S. mitis* (2 bcs)	Thickening valve leaflet	Severe valve regurgitation	N		N	N	N	
49	On antibiotics	Small mobile element (degenerative)	Prosthetic valve, CIED	N		N	N	N	
50	Bacteremia *S. epidermidis* (3 bcs)	Small mobile element (degenerative)		N		N	N	N	

51	Bacteremia *A. defectiva* (2 bcs)	Valve vegetation		Y		N	N	N
52	On antibiotics	Abnormal metabolic activity (^{18}F-FDG PET/CT) of CIED-lead	CIED	N		N	N	N
53	Bacteremia *P. aeruginosa* (2 bcs)	Abnormal metabolic activity (^{18}F-FDG PET/CT) of CIED-lead	CIED	Y		N	N	N
54	Bacteremia *S. agalactiae* (1 bcs)	Small mobile element (degenerative)	Prosthetic valve, CIED	Y		N	N	N
55	Bacteremia (community-acquired) *E. faecium* (3 bcs)	Abnormal metabolic activity (^{18}F-FDG PET/CT) of CIED-lead	CIED	N		N	N	N
56	Bacteremia *S. epidermidis* (2 bcs)	Small mobile element (degenerative)		Y	Pulmonary septic emboli	N	N	N
57	On antibiotics	Small mobile element (degenerative)	Severe valve regurgitation	N		Y	Y	Y
58	Bacteremia *S. aureus* (2 bcs)	Small mobile element (degenerative)	Severe valve regurgitation	N		N	N	N
59	On antibiotics	Abnormal metabolic activity (^{18}F-FDG PET/CT) of prosthetic valve	Prosthetic valve, CIED	Y		N	N	N
60	On antibiotics	Abnormal metabolic activity (^{18}F-FDG	Prosthetic valve, CIED	N		N	N	N

		PET/CT) of prosthetic valve						
61	On antibiotics	Abnormal metabolic activity (^{18}F-FDG PET/CT) of prosthetic valve	Prosthetic valve, CIED	N	Cerebral emboli, renal emboli	N	N	N
62	Bacteremia *Gemella spp.* (3 bcs)	Abnormal metabolic activity (^{18}F-FDG PET/CT) of prosthetic valve	Prosthetic valve, CIED	Y		N	N	N
63	Bacteremia *S. aureus* (2 bcs)	CIED-lead lesion (degenerative)	CIED	N		N	N	N
64	On antibiotics	CIED-lead lesion (degenerative)	Prior IE, CIED	N		N	N	N
65	Bacteremia *S. aureus* (2 bcs)	CIED-lead lesion (degenerative)	Prior IE, CIED	N		N	N	N
66	On antibiotics	Valve vegetation	Prior IE, CIED	N		N	N	N
67	On antibiotics	CIED-lead vegetation	CIED	N		N	N	N
68	On antibiotics	CIED-lead vegetation	Prior IE, CIED	N		N	N	N
69	On antibiotics	Valve vegetation		N		N	N	N
70	On antibiotics	Small mobile element (degenerative)	Prosthetic valve, CIED	Y		Y	Y	N
71	Bacteremia (community-acquired) *E. faecalis* (3 bcs)	Small mobile element (degenerative)	Prior IE, CIED	Y		N	N	N
72	Bacteremia *S. epidermidis* (3 bcs)	CIED-lead lesion (degenerative)	CIED	Y		N	N	N

73	Bacteremia *S. lugdunensis* (3 bcs)	Valve vegetation, abscess	TAVI	N	Y	Y	N
74	Bacteremia *S. epidermidis* (2 bcs)	CIED-lead vegetation	CIED	N	N	N	N

^{18}F-FDG PET/CT: ^{18}F-Fluorodeoxyglucose Positron Emission Tomography/Computed Tomography; bcs: blood culture set; CIED: cardiac implantable electronic device; IE: infective endocarditis; N: no; TAVI: Transcatheter aortic valve implantation; Y: yes

Supplementary Figure 1. Classifications of episodes by different versions of Duke criteria based on **A)** clinical criteria, **B)** clinical criteria and implementation of the pathological confirmation and rejection criteria and **C)** final infective endocarditis diagnosis.

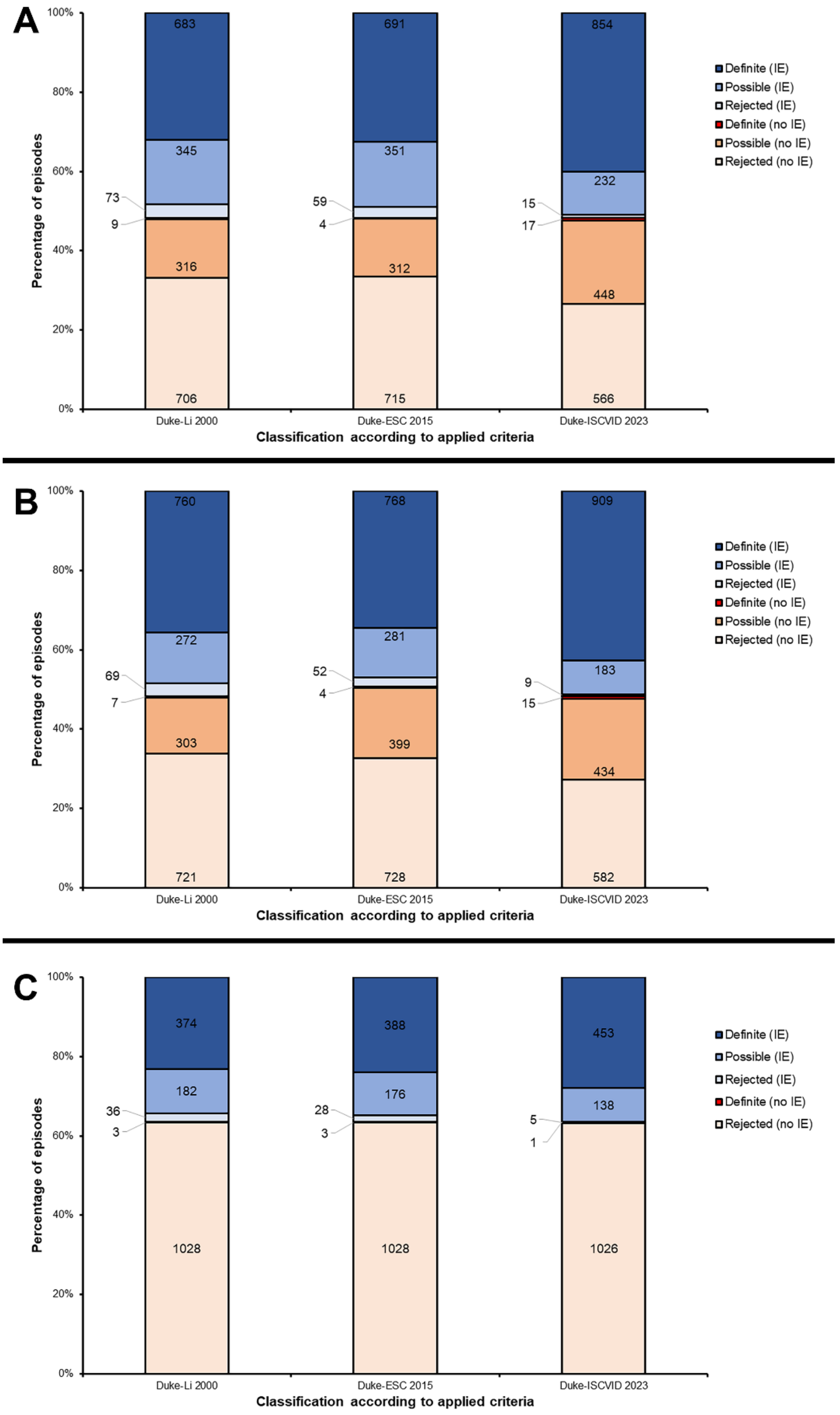

Supplement: ciae039_Supplementary_Data [file ciae039_supplementary_data.pdf]
